# A Dislodged Tooth Replaced

**Published:** 1868-01

**Authors:** H. H. Keech


### ARTICLE V.

### *A Dislodged Tooth Replaced.*

By H. H. Keech, D.D.S.

On the 11th, of October, 1864, Mrs. M.——of this city called on me with her son ten years of age, who while playing in the street fell on the curb-stone and knocked his left superior central incisor entirely out. I saw him about half an hour after the accident occurred. The first thing I did was to place the tooth in luke warm water. I then syringed the cavity with warm water, though it had bled very little, and there was scarcely any clotted blood in it. I then placed strong silk on the tooth, and forced it up into its socket, the cutting edge a little above the adjoining teeth, and requested his mother to hold it there while I made fast the silk. The pain caused by putting the tooth in was very severe, and made the perspiration come out in great drops on the little fellow's forehead. I directed the frequent use of Harris' gum wash and laudanum, kept the silk on two weeks, have seen the tooth frequently since, and it seems as perfect as any in his head, and abscess has never occurred, nor is it the least discolored on the labial surface, though you can by looking very closely see the slightest discoloration on the palatine surface. Should the tooth become dark, I will drill in, bleach, and fill to the end of the root.

The question may be asked by some, why was not this tooth drilled and filled before it was replaced as it could have been done very conveniently? I answer, by saying, that it could not have been done without destroying the periosteum which was attached to the tooth, thereby rendering the tooth, by the loss of this periosteum, entirely a dead body. The periosteal attachment is now as firm as it ever was, and the tooth as perfect as any tooth can be when the nerve is dead. This case should have been recorded sooner, had it not been that I wished to wait until I could say it was an entire success.

### SELECTED ARTICLES.

